# Correlation of SVINT and Sensory Organization Test in Children with Hearing Loss

**DOI:** 10.3390/audiolres12030033

**Published:** 2022-06-06

**Authors:** Solara Sinno, Fadi Najem, Georges Dumas, Kim Smith Abouchacra, Art Mallinson, Philippe Perrin

**Affiliations:** 1 EA 3450 DevAH, Development, Adaptation and Handicap, Faculty of Medicine, University of Lorraine, 54500 Vandoeuvre-lès-Nancy, France; georges.dumas10@outlook.fr (G.D.); philippe.perrin@univ-lorraine.fr (P.P.); 2 Laboratory for the Analysis of Posture, Equilibrium and Motor Function (LAPEM), University Hospital of Nancy, 54500 Vandoeuvre-lès-Nancy, France; 3 Department of Audiology, University of the Pacific, San Francisco, CA 95211, USA; fadinajem2000@yahoo.com; 4 Department of Oto-Rhino-Laryngology, Head and Neck Surgery, Grenoble Alpes University Hospital, 38043 Grenoble, France; 5 Department of Otorhinolaryngology-Head and Neck Surgery, Audiology & Balance Center, American University of Beirut Medical Center, Beirut 1107 2020, Lebanon; ks05@aub.edu.lb; 6 Division of Otolaryngology, Faculty of Medicine, University of British Columbia, Vancouver, BC V6T 1Z4, Canada; artmallinson@gmail.com; 7 Department of Pediatric Oto-Rhino-Laryngology, University Hospital of Nancy, 54500 Vandoeuvre-lès-Nancy, France

**Keywords:** hearing loss, children, skull vibration-induced nystagmus, posturography, caloric test, video head impulse test

## Abstract

*Objective*: The skull vibration-induced-nystagmus test (SVINT) is a noninvasive and effective screening tool for the function of the otolith and canal structures in children. It can instantaneously assess vestibular asymmetry. This study aimed to analyze the SVINT results of healthy children vs. children with hearing loss (HL) and to correlate it with sensory organization test (SOT) results as a functional balance evaluation tool. *Design*: This case-controlled study compared the results of SVINT to the results of the SOT of the computerized dynamic posturography (CDP) in a control group of 120 healthy normal-hearing children (i.e., NH group) vs. hearing loss (HL) group of 60 children, including 30 children with hearing aids (HAs) and 30 children with a unilateral cochlear implant (CI). The SVINT results were compared to the caloric test (CaT) and video head impulse test (vHIT) and associated with SOT scores. *Results*: Thirty-one children in the HL group had normal SVINT and normal SOT results. A total of 21 children in the HL group had SVINT-negative and abnormal results in the SOT (possibly due to bilateral vestibular loss (BVL)). Eight children in the HL group had positive SVINT and abnormal SOT results. However, none of the children had only positive SVINT with normal SOT findings. Moreover, 52% of children had a normal result on both the SOT and CaT, whereas 27% had abnormal results on both tests (17% bilateral weakness and 10% unilateral), and 22% had the only result of the SOT suggesting a functional abnormality. Similarly, when associating the result to vHIT, 51% had normal results on both tests, and 25% had abnormal results (13% bilateral and 12% unilateral weakness). *Conclusions*: SVINT findings can be correlated with SOT findings in the case of the unilateral vestibular lesion (UVL), which adds a diagnostic value in these pediatric cases but may differ in the case of the bilateral vestibular lesion (BVL). However, SVINT findings need to be cautiously interpreted in light of other test findings such as the SOT, CaT, and vHIT.

## 1. Introduction

To maintain balance and posture, the central nervous system integrates sensory information from different components of the vestibular, the visual, and the somatosensory systems. Postural control responds to the changes in the environmental input to maintain the body’s center of gravity within the base of the postural support [1]. The postural control ability changes with age. It improves at a younger age due to the maturation and development of different sensory systems. The ability to control posture relies first on proprioceptive inputs, second on visual, and lastly on vestibular inputs, which are the latest to mature [2], but it gradually declines later in life. Moreover, the weighting of sensory inputs, the reliance on distinct strategies to maintain balance, and the integration between different strategies change across the life span [3]. Postural stability analyses of different strategies and sensory interactions are carried out by the Sensory Organization Test (SOT). The SOT is one of the test batteries included in the Computerized Dynamic Posturography (CDP). The SOT can objectively identify the various dysfunctions of the visual, vestibular, and somatosensory systems by forcing the reliance on one sensory input (e.g., vestibular) to maintain postural control while isolating the other sensory inputs (e.g., vision and somatosensory). The sensory organization test (SOT) assesses the overall balance and the use of specific sensory inputs to maintain postural control.

In some children with congenital hearing loss, associated vestibular symptoms have been observed. This is probably because the inner ear encloses the anatomical structures of the auditory and vestibular systems. There is an embryological and anatomical correlation between the cochlea and the vestibular system. In deaf children, congenital or acquired vestibular dysfunction can occur in addition to hearing loss, leading to balance impairment [4,5]. Therefore, assessing the vestibular function in deaf children can be important and recommended in some cases. Hearing aids (HAs) and cochlear implants (CIs) helped in changing the quality of life of many people with hearing impairment. Borsetto et al. showed in a systemic review that the static and dynamic subjective perception of balance may improve in adult patients after using HAs, but the mechanism and the extent of benefit are still unclear [6]. It is still unclear if HAs can improve pre-existing vertigo or worsen it in children. 

Some children might show vestibular dysfunction symptoms post-CI surgery. In these cases, a vestibular evaluation becomes more essential before and after an invasive procedure for rehabilitating hearing loss, such as CI surgery. Pediatric vestibular evaluation requires the use of sufficiently reliable methods that are simple and noninvasive to be used with the pediatric population. To study the integrity of the vestibular system in pediatrics, various tests have been performed and reported in the literature, such as the caloric test (CaT) that provides information about the lateral semicircular canals (SCCs) for each side and the oculomotor test that explores the central nervous system. Moreover, technological advancements provided clinicians with new methods to explore the vestibular function in the pediatric population such as the video head impulse test (vHIT) that evaluates the function of the three SCCs for each side and the vestibular-evoked myogenic potential (VEMP) that evaluates the otolithic function. The skull vibration-induced nystagmus test (SVINT) (also known as the Dumas test) is considered a fast, noninvasive, and effective method for evaluating vestibular dysfunction. Previous data from large cohorts of adult and pediatric patients within the last ten years showed that SVINT can be effective in evaluating the different age groups of patients and those with compensated vestibular dysfunction. The SVINT can detect interaural vestibular asymmetry, and it is performed by observing the patient’s eyes for nystagmus while applying a 100 Hz vibratory stimulus to the patient’s skull (usually on the mastoid bone) for a short period (5–15 s) [7]. The SVINT has been demonstrated as a rapid, noninvasive test in children [8] and is mainly relevant to the contribution of the superior branch of the VIII cranial nerve [9,10]. The SVINT is mainly relevant to the contributions of the SCC and the utricle, but not to the contribution of the saccule [8].

The clinical significance of using the SVINT in evaluating vestibular function in pediatrics is still not fully established and requires more empirical investigation. Therefore, the present study examines the correlation between the SVINT and the SOT findings in normal-hearing pediatric subjects vs. hearing-impaired pediatric subjects who are rehabilitated with HAs and CIs. Keeping in mind that performing the gold standard caloric test is challenging in the pediatric population, the aim of the present study is to explore the clinical diagnostic value of using SVINT in identifying unilateral and asymmetrical vestibular lesions in children with hearing loss by correlating the SVINT results with the SOT results and with the vHIT and CaT. 

## 2. Participants and Methods

This case-control study was conducted over a period of 24 months after approval from the Institutional Review Board at the American University of Beirut, Lebanon (OTO.KA.05 and 07). All tests were performed in the Audiology and Balance Center at the American University of Beirut Medical Center (AUBMC) and were performed by the same examiner. Written consent was obtained from the primary caregiver and the child before starting the test. 

### 2.1. Participants

Participants were divided into two groups in this case-control study: the control group (NH group) included 120 healthy, normal-hearing subjects, and the hearing-impaired group (HL group) included 30 hearing-impaired children who used HAs and 30 hearing-impaired children who use CIs unilaterally. All children were between 5 and 17 years of age. The average age was 11.33 (±3.57) years for the NH group, 12.23 (±3.58) years for the HA users, and 9.17 (±3.01) years for the CI users. Table 1 summarizes the gender and age details of the different age ranges. The etiology of hearing loss was not the same across the groups: The main etiology of hearing loss in children with HAs was due to genetic factors, whereas idiopathic etiology was the highest reported cause of hearing loss, followed by genetic factors and then meningitis in children with CIs. The level of hearing loss was between moderate–severe (56–70 dB) and severe (71–90 dB) bilaterally for children with HAs and bilaterally profound for children with CIs.

### 2.2. Equipment

For SVINT, the eye movements were recorded using the VNG Ulmer device (Synapsys, Marseille, France) while using a vibrator VVIB 100 (Synapsys, Marseille, France). The vHIT was recorded using the ICS Impulse (GN Otometrics, Denmark). For the standard sensory organization test (SOT) evaluation, a SMART Equitest posturography system (Natus; NeuroCom International, Clackamas, OR, USA) was used.

### 2.3. Procedure

The NH group included normal-hearing children who did not have any history of otological or neurological disorders (e.g., recurrent otitis media, dizziness, etc.), speech–language delays, or mental deficits. Children with ocular or ophthalmological pathologies, abnormal oculomotor results, abnormal caloric responses, or spontaneous nystagmus were excluded from the study. The HL group included children with sensorineural hearing loss (SNHL) who use HAs or CIs. Children with other syndromic pathologies, neurological complaints, or mental deficits were excluded from the study.

The following procedure was used in previous publications by the authors about SVINT [2,8,11]. Each child underwent an age-appropriate audiological evaluation including otoscopy, pure tone audiometry, and immittance. Pure tone audiometry was performed using the (Grason-Stadler Audiostar pro audiometer), and the immittance testing was performed using the Grason-Stadler Tympstar pro tympanometer).

SVINT was performed using the vibrator VVIB100, which was applied firmly and perpendicularly on 3 positions successively: the vertex and the right and the left mastoid processes at the level of the external auditory canal for 5 to 20 s at each position. The eye movement was recorded during each vibration position to identify the presence or absence of nystagmus.

CaT assessed the vestibulo–ocular reflex (VOR) of the horizontal canal function (at a very low frequency) by irrigating cold and warm air in the external ear canal. Caloric testing was performed following the British Society of Audiology recommendations. In the group of normal children, the right and left ears were irrigated for 60 s with cold air (24 °C). The child was excluded from the study in the case of asymmetrical cold caloric findings. For children with HL, the right and left ears were irrigated with warm and cold air (24–50 °C). A mental task was given to all children during the caloric test to ensure a maximum intensity and regularity of the nystagmus response. An interval of at least 5 min was given between irrigations allowing the child to rest and ensuring the absence of temperature influence on the following contralateral irrigation. The results of the CaT were analyzed in the categorical format, and the asymmetry was calculated from maximum slow phase velocities using Jongkees’ formula. The monothermal caloric asymmetry (MCA) criteria for unilateral weakness (UW) was defined as >15%, and the total bilateral reflectivity for bilateral weakness (BW) was defined as <12°/s according to the British Society of Audiology guidelines [12].

vHIT measured the velocity of the eyes and recorded abnormalities in patients with VOR dysfunction at high frequencies of 4–7 Hz. The right and left lateral SCC vHIT findings were only analyzed for the present study.

SOT assessed the overall balance and the use of specific sensory inputs to maintain postural control. This included the 6 common conditions: (1) eyes open, surround and platform stable, (2) eyes closed, surround and platform stable, (3) eyes open, sway-referenced surround, platform stable, (4) eyes open, sway-referenced platform, (5) eyes closed, sway-referenced platform, and (6) eyes open, sway-referenced surround and platform. The composite equilibrium scores (CES) and the sensory analysis ratios (SASs) were quantified and compared among the 6 conditions as follows:

- The somatosensory ratio compared Condition 2 to Condition 1 and assessed the ability of an individual to use somatosensory information for balance.
- The visual ratio compared Condition 4 to Condition 1 and assessed the ability of an individual to use visual information for maintaining balance.
- The vestibular ratio compared Condition 5 to Condition 1 and assessed the ability of an individual to use vestibular information for maintaining balance.
- The visual preference ratio compared Conditions 3 and 6 to Conditions 2 and 5 and assessed the degree to which an individual relied on visual information to maintain balance, even when the information was incorrect or misleading.

### 2.4. Statistical Analysis

The collected data were statistically analyzed using SPSS software (v25.0, IBM, Chicago, IL, USA). The null hypothesis assumes no correlation between the SVINT and the SOT findings and no significant difference in the SVINT and SOT results between the control group and the HL group. The means and standard deviations (SDs) were used to describe the study sample. Multiway Analysis of Variance (MANOVA) was used to analyze the significance of the main effects of (2 experimental groups: NH vs. HL groups) × (2 tests (SVINT vs. SOT)) × (4 age ranges (5–8 vs. 9–11 vs. 12–14 vs. 15–17 years)) × (2 Genders (Male vs. Female)).

The results of the audiological evaluation, SVINT, CaT, and SOT were transformed into categorical data as shown in Table 2. Chi-square tests (X^2^) and Fisher’s exact tests (F) were used to compare the main effects across groups using a significance level of *p* < 0.05. 

## 3. Results 

### 3.1. SVINT

Among the 120 healthy children in the NH group, three children (2.5% of cases) showed nystagmus with slow phase velocity < 2.5°/s during SVINT. These rare positive SVINT findings in normal subjects have been reported in the literature [11,13] and can be overlooked here because the nystagmus magnitude was marginal and does not statistically affect the findings of the present study. A negative SVINT result was observed in the remaining 117 children (97.5%) according to the criteria defined in Table 2. Among these children with negative SVINT, 94 children did not have any nystagmus, and 23 children had nonclinically significant nystagmus (For more details, please review the full data published in SVINT as a screening tool in children [14]). In children, SVINT can detect unilateral vestibular deficit in a very high frequency with a sensitivity of 86% and a specificity of 96%. The positive predictive value is 75%, and the negative predictive value is 98% [14].

### 3.2. Caloric Test 

Children in the NH group had to have a normal CaT and vHIT to be included in the study. Seventeen percent of cases in the HL group had bilateral hyporeflexia, and 10% had unilateral hyporeflexia. The X^2^ revealed that children using hearing aids are more prone to bilateral vestibular lesions (X^2^ = 43.6 and *p* = 0.001). The effects of age and gender were insignificant (FET = 5.62 and *p* = 0.45) and (FET = 4.13 and *p* = 0.13) for age and gender, respectively.

### 3.3. vHIT Test

Children in the HL group showed 13% bilateral weakness and 12% unilateral weakness in the horizontal canal tests. In the present study, the results of this test were not affected by age (FET = 9.58 and *p* = 0.29) or gender (FET = 2.27 and *p* = 0.59).

### 3.4. SVINT vs. CaT

**Figure 1:**
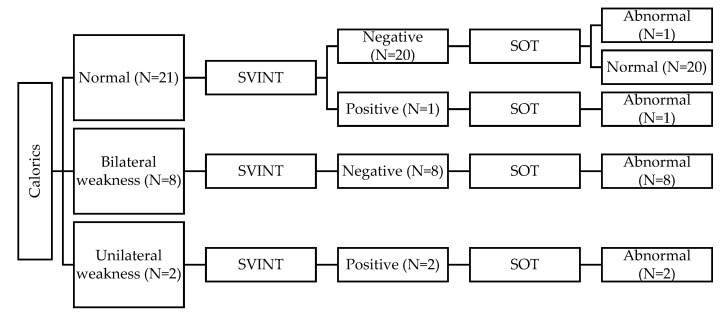
Summary of results for children with hearing aids (HAs).

**Figure 2:**
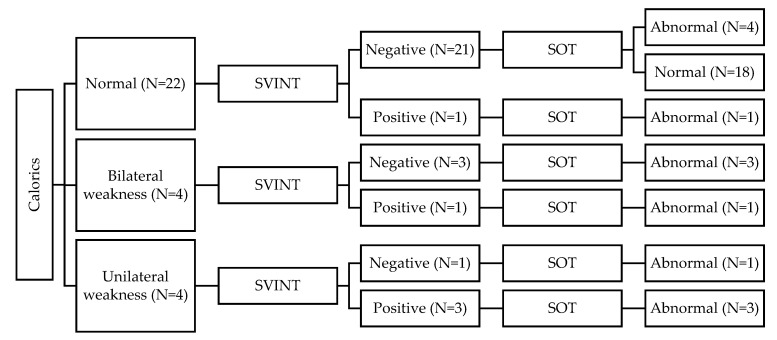
Summary of results for children with cochlear implants (CIs).

When taking the CaT as the gold standard and comparing the findings to the SVINT, the SVINT showed a high correlation with the CaT (FET = 18.28 and *p* = 0.001). Forty-two out of the 60 children in the HL group (70%) had normal CaT and SVINT results bilaterally. Ten children had abnormal CaT findings but had normal SVINT findings. Nine of these children showed bilateral weakness (BW), and one child showed unilateral weakness (UW). Six children had abnormal results in both tests. Five of these children showed UW in both CaT and SVINT, and one child showed BW in the CaT vs. UW on SVINT. Two children had normal CaT vs. abnormal SVINT (i.e., UW). Figure 1 and Figure 2 summarize the results of CaT and SVINT in the HL group for children with HAs and children with CIs, respectively.

### 3.5. SVINT vs. vHIT

Similarly, SVINT correlated with the vHIT results (FET = 24.04 and *p* = 0.001). Forty-two children in the HL group showed normal results on both tests (71%). Six children (10%) had both tests showing UW. The vHIT results showed BW in eight children with normal SVINT results and showed UW in one child with normal SVINT results. Two children showed abnormal SVINT results; one child showed BW on the vHIT result, and one child showed normal vHIT.

### 3.6. SVINT vs. SOT

In reference to the SOT normative data of the NH group that was previously published [2], a comparison to the data obtained from the HL group was conducted by matching the age and gender of the cases. For children with HA, the nonparametric Kruskal–Wallis test showed a significant improvement in the composite score around 11 years old (t = 15.68, df = 3, and *p* = 0.001). The somatosensory ratio was the same in all age ranges (*p* = 0.778). The visual ratio showed that the maturation was divided into two groups (aged 5 to 8 years and 9 to 11 years vs. 12 to 15 years and 15 to 17 years) (t = 11.178, df = 3, and *p* = 0.011). A slight but insignificant improvement in the mean vestibular ratio was observed in the older age range (*p* = 0.305). The visual preference score was significantly lower in the 5 to 8 age range compared to the 9 to 11 age range (t = 10.03, df = 3, and *p* = 0. 01). The visual preference score for the 9 to 11 age range was similar to those of the two older age ranges. For children with CIs, the Kruskal–Wallis nonparametric test showed that the composite score increased with age (t = 12.76, df = 3, and *p* = 0.005). The somatosensory ratio was not affected by age. The visual ratio showed improvement with age (t = 9.278, df = 3, and *p* = 0.026). The vestibular performance was not influenced by age (*p* = 0.734). However, the visual preference peaked at 12 to 14 years (t = 8.31, df = 3, and *p* = 0.04). In the HL group, gender did not affect the composite score nor the sensory ratios.

In all age ranges, the composite score was higher in the NH group than in the HL group (F 5–8 years = 473, *p* = 0.001; F 9–11 years = 372, *p* = 0.001; F 12–14 years = 333, *p* = 0.009; and F 15–17 years = 264, *p* = 0.019, respectively). The visual ratio was higher in the HL group for children aged 15 to 17 years (F = 86 and *p* = 0.008). The visual preference ratio was lower in the HL group for children aged 5 to 8 years (F = 485 and *p* = 0.001) and children aged 9 to 11 (F = 353 and *p* = 0.002). 

The SVINT results correlated with the SOT results (FET = 9.86 and *p* = 0.002). A correlation was noted between the SVINT, on one hand, and the vestibular ratio (Mann–Whitney = 316 and *p* = 0.019) and the composite score (U = 297 and *p* = 0.05) of the CDP, on the other hand. The somatosensory ratio, visual ratio, and visual preferential ratio did not show any significant correlations with the SVINT findings (*p* ≥ 0.05). 

### 3.7. SOT vs. CaT

When comparing the SOT results to the CaT results, 31 children had normal results on both tests. Twenty-nine children had abnormal results in the SOT. Thirteen out of these children had normal CaT; 10 had BW, and 6 had UW (*p* = 0.001) (Table 3).

### 3.8. SOT vs. vHIT

When comparing the SOT results to the vHIT results, 30 children had normal results in both tests. Thirteen had abnormal results in the SOT even though the vHIT was normal (*p* = 0.001) (Table 3).

## 4. Discussion

The anatomical and physiological proximity of the cochlea to the peripheral vestibular system can result in vestibular dysfunction in some cases of sensorineural hearing loss. Dysfunction of the vestibular end organs can interfere with the ability to maintain static and dynamic equilibrium. In 1955, Arnvig concluded that the prevalence of vestibular dysfunction in hearing-impaired children varied between 20% and 70% [15]. Rosenblüt et al. (1960) studied vestibular function in 107 deaf children and noted the very important correlation between vestibular and hearing dysfunctions. However, this link cannot be generalized and remains case-dependent [16]. Likewise, Sandberg and Terkildsen (1965), who tested 57 hearing-impaired children, reported a possible correlation between hearing loss and vestibular function, but they could not conclude this correlation with a high confidence level. They postulated that the vestibular function continues to be normal until the hearing function is profoundly or entirely lost [17]. 

In the present study, 45% of children in the HL group had abnormal findings of at least one of the conducted vestibular tests (i.e., SOT, CaT, and SVINT). This prevalence percentage reported in the present study (45%) agrees with the prevalence of vestibular dysfunction in hearing-impaired children reported by Arnvig in 1955 (20% to 70%) [15]. Also, the findings show that the link between vestibular dysfunction and hearing loss can be established but has to be investigated on individual bases as recommended previously by Rosenblüt et al. (1960) and Sandberg and Terkildsen (1965) [16,17]. 

Different researchers have used various methods and/or clinical examinations to evaluate vestibular function in hearing-impaired patients. For example, Selz et al. exploited oculomotor tests in hearing-impaired children, and they reported prolonged latencies of saccadic eye movements in these children [18]. In other words, oculomotor tests were not the ideal tests for evaluating balance dysfunction in hearing-impaired children. The “gold standard” for evaluating the balance function in pediatrics that is reported in the literature is the CaT. Brookhouser and Cyr tested 166 deaf children, and they found that 10% of the children had unilateral labyrinthine hypofunction, and 12% had bilateral labyrinthine hypofunction in response to caloric stimulation. They concluded that the extent of the hearing loss is not a useful predictor of caloric hypoactivity [19]. 

In the present study, the examination method was extended to include different methods including the SVINT, CaT, SOT, and vHIT. The findings show interesting results for each of these tests. Twenty percent showed bilateral weakness detected by the CaT only; 13.3% showed unilateral weakness detected by the CaT and SVINT; 11.7% showed vestibular dysfunction detected by the SOT only, and 25% had abnormal results (13% bilateral and 12% unilateral weakness) detected by the vHIT. Other vestibular assessment methods were not investigated in the present study, such as VEMP. However, previous studies showed that VEMP findings can be valuable in evaluating vestibular function in this population (children with hearing loss). Cushing et al. reported that 50% of 40 hearing-impaired children had lateral SCC hypofunction, and 38% had upper SCC dysfunction, and 40% had functional abnormalities tested with VEMP [20,21]. Despite their demonstration of normal peripheral vestibular function based on an appropriate vestibulo–ocular reflex during the rotation test, their group of participants may have had central sensory organization abnormalities that caused instability under all conditions of SOT [22]. 

In the present study, the SVINT vs. CaT correlation data show that 10 HL children had abnormal CaT findings but had normal SVINT findings. Nine of these children showed bilateral weakness (BW), and one child showed unilateral weakness (UW). Six children had abnormal results bilaterally. Five of these children showed UW in both CaT and SVINT, and one child showed BW in the CaT vs. UW on the SVINT. Two children had normal CaT vs. abnormal SVINT (i.e., UW). Similarly, the SVINT vs. vHIT correlation data showed that six HL children (10%) had both tests showing UW. The vHIT results showed BW in eight children with normal SVINT results and showed UW in one child with normal SVINT results. Two children showed abnormal SVINT results; one child showed BW on the vHIT result, and one child showed normal vHIT. Finally, the SVINT vs. SOT correlation data showed that SVINT results correlated with the SOT results (FET = 9.86 and *p* = 0.002). A correlation was noted between the SVINT, on one hand, and the vestibular ratio (Mann–Whitney = 316 and *p* = 0.019) and the composite score (U = 297; *p* = 0.05) of the CDP, on the other hand. These findings clearly show the valuable clinical value of using SVINT to demonstrate unilateral and/or asymmetrical bilateral vestibular dysfunction in HL children. On the other hand, these findings showed the limited diagnostic value of SVINT in diagnosing bilateral symmetrical vestibular disfunction. The findings of the present study in combination with the previous literature should enlighten the path toward more comprehensive and practical protocols to evaluate the vestibular function in the pediatric population, especially those with hearing loss. SVINT can add a simple, rapid significant clinical value in the diagnosis of unilateral or asymmetrical vestibular disfunction. However, the findings of the present study show that SVINT findings must be interpreted in light of other test results, such as CaT, SOT, vHIT, etc. Finally, the findings show, as expected, the limited clinical value of SVINT in diagnosing bilateral symmetrical weaknesses. 

Crowe and Horak conducted a cross-sectional study, and their findings showed that many children with hearing loss had poor balance and motor skills [23]. Likewise, Hartman et al. examined the response latencies to destabilizations in deaf primary school children and found that their performance in these motor skills was slower than their healthy peers in the control group [9]. Potter and Silvermann reported that many deaf children compensated for vestibular deficits through the visual and somatosensory systems to maintain static balance with eyes open or closed [10]. The findings of the present study add valuable data to these previous reports of a possible correlation between hearing loss and vestibular dysfunction in the pediatric population. There is a dearth in the literature regarding different methods of evaluating vestibular function in the pediatric population, and thus, more research is needed to enhance the clinical procedures and the diagnostic accuracy of assessing vestibular lesions in children. 

The findings of the present study demonstrate the importance of the SOT in detecting BVL as well as the importance of SVINT in detecting UVL and in detecting asymmetry in bilateral lesions. The present study also provides correlation findings between the SVINT, on one hand, and the CaT, vHIT as well as the vestibular ratio and the composite score of the SOT, on the other hand. These correlation findings are important and should be considered in future clinical protocols for evaluating vestibular function in children because they provide the opportunity to cross-check between these clinical tests to improve the diagnostic accuracy of the vestibular dysfunction in children.

Finally, all children in both (NH and HL) groups denied any change in hearing, any discomfort, any changes in the performance of the HAs or CIs, or any other negative effects at the end of data collection. Therefore, one can conclude that all the procedures used in the present study including the SVINT were safe and had no adverse effect on the children or their devices. Similar safety concerns were addressed and dissipated in the previous literature [14].

## 5. Conclusions

The previous literature on the subject suggested that the SVINT shows a robust sensitivity in patients with UVL and shows a high correlation with CaT. However, it is less sensitive in bilateral symmetrical deficits, where the SOT is a better evaluation tool in these cases. The findings of the present study show that SVINT can be correlated with the SOT in the case of the UVL, which adds a diagnostic value in these pediatric cases but cannot be correlated with the SOT in the case of the BVL. Moreover, the SVINT findings need to be cautiously interpreted in light of other test findings, such as the SOT, CaT, and vHIT. According to the findings of the present study, SVINT can be used as an additional and valuable clinical tool to confirm UVL and asymmetrical BVL in pediatrics. However, in cases of bilateral symmetrical weakness and for follow-up purposes with the physical therapist, the SOT remains the test of choice to reveal the bilateral balance deficit and to monitor the rehabilitation outcome progress.

## Figures and Tables

**Table 1:** Descriptive demographics of the groups of children by age, gender, and hearing status.

Age Range (in Years)	NH Group (Control)			HL Group (HA Users)			HL Group (CI Users)		
	Female	Male	Total	Female	Male	Total	Female	Male	Total
5–8	15	15	30	2	3	5	7	6	13
9–11	15	15	30	4	1	5	5	5	10
12–14	15	15	30	5	5	10	3	2	5
15–17	15	15	30	5	5	10	1	1	2
Total	60	60	120	16	14	30	16	14	60

**Table 2:** Vestibular assessment normal criteria.

Assessment Procedure	Criteria for Normal Findings	Criteria for Abnormal Findings
*Audiological evaluation*	**NH Group:** Hearing thresholds lower than 15 dB across the octave frequencies from 250–8000 Hz bilaterally. Normal middle ear function (Type A tympanogram with present ipsilateral and contralateral acoustic reflexes bilaterally.	**HL Group:** Children using HAs: Moderate–severe (56–70 dB) and severe (71–90 dB) hearing loss. Children using unilateral CIs: had profound hearing loss (>90 dB).
*Skull vibration-induced nystagmus* (*SVINT*)	**Negative SVINT:** No nystagmus is recorded, or the observed nystagmus is less than 10 beats per second (i.e., slow velocity phase (SPV) < 2°/s) in 1 or 2 locations or changing its direction.	**Positive SVINT:** Vestibular asymmetry seen in cases of horizontal/rotary nystagmus was observed (more than 10 beats and SPV > 2°/s) in two or more locations, beating toward the same direction, and it is reproducible in at least 2 locations.
*Caloric Test* (*CaT*)	Robust and equal response from both ears. Symmetry was measured using the Jongkees’ formula and by following the standards recommended by the British Society of Audiology.	Unilateral weakness exceeding 15% or bilateral weakness with total reflectivity of responses from both sides less than 12°/s.
*Video Head Impulse Test* (*vHIT*)	Vestibular–ocular reflex (VOR) gain greater than 0.7 for the lateral semicircular canals.	Unilateral lateral SCC abnormality: One lateral canal with VOR gain greater than 0.7 and one lateral SCC with a VOR gain lower than 0.7. Bilateral lateral SCC abnormality: Bilateral VOR gain is lower than 0.7.
*Posturography*	The composite equilibrium score (CES) measures the overall level of performance on the SOT and is a weighted average calculated from the mean performance of the ES of Conditions 1 and 2 and the average from Conditions 3 through 6; it should be within the normal range for each of the equilibrium scores (ESs) based on age and gender.	Lower CES or fall for each of the 6 conditions is considered abnormal results.

**Table 3:** Correlation of posturography (SOT) between the caloric test (CaT) and video head impulse test (vHIT).

	Caloric Test			
	Normal	Bilateral Weakness	Unilateral Weakness	Total
SOT Normal	31	0	0	31
SOT Abnormal	13	10	6	29
Total	44	10	6	60
	**Video Head Impulse test**			
	**Normal**	**Bilateral Weakness**	**Unilateral Weakness**	**Total**
SOT Normal	30	0	0	30
SOT Abnormal	13	8	7	28
Total	43	8	7	59

## Data Availability

Not applicable.
